# Plant-Derived Polyphenols in Cancer Therapy: Bridging Molecular Mechanisms and Bioavailability Toward Clinical Translation

**DOI:** 10.3390/pharmaceutics18060737

**Published:** 2026-06-13

**Authors:** Syed Arman Rabbani, Shrestha Sharma, Mohamed El-Tanani, Suman Khurana, Manita Saini, Monu Yadav, Rakesh Kumar, Yahia El-Tanani

**Affiliations:** 1 RAK College of Pharmacy, Ras Al Khaimah Medical and Health Sciences University, Ras Al Khaimah P.O. Box 11172, United Arab Emirates; 2 Amity Institute of Pharmacy, Amity University, Gurugram 122413, India; 3 Department of Pharmacy, Panipat Institute of Engineering and Technology (PIET), Panipat 132102, India; 4 Geeta Institute of Pharmacy, Geeta University, Panipat 132145, India; 5 Department of Pharmacy, Jagannath University, Bahadurgarh 124507, India; 6 Royal Cornwall Hospital Trust, NHS, Truro TR1 3LJ, UK

**Keywords:** cancer, plant-derived polyphenols, clinical translation, anticancer efficacy, bioavailability, molecular mechanisms, nanotechnology, next-generation cancer therapeutics

## Abstract

Cancer is still one of the world’s major causes of morbidity and mortality; thus, safer and more efficient treatment approaches are required. The structural variety, multitargeted mechanisms, and generally good safety profiles of plant-derived polyphenols have made them attractive anticancer medicines. Flavonoids (like quercetin), stilbenes (like resveratrol), phenolic acids and curcuminoids (like curcumin) are major classes that have shown strong anticancer action against a variety of cancers, including prostate, colorectal and breast cancers. Through targets including PI3K/Akt, MAPK, NF-κB, and p53 signaling networks, these substances influence important molecular pathways involved in tumor initiation and development, including oxidative stress, inflammation, apoptosis, cell cycle control, angiogenesis and metastasis. The clinical translation of polyphenols is still constrained by poor bioavailability, fast metabolism, low aqueous solubility and inefficient pharmacokinetic characteristics, which lead to insufficient systemic exposure and therapeutic efficacy despite strong preclinical data. Their therapeutic applicability is further complicated by variations in absorption and possible dose-related restrictions. To overcome these limitations, the anticancer efficacy of polyphenols has been enhanced via delivery technologies like polymeric nanoparticles, lipid-based carriers, nanoemulsions and phytosome complexes, which have shown improved stability, increased bioavailability and targeted delivery to tumor tissues. This review provides a comprehensive and integrative analysis of plant-derived polyphenols by linking molecular mechanisms, pharmacokinetic limitations and emerging delivery strategies within a translational framework. By bridging these interconnected domains, this review highlights the potential of polyphenols as viable candidates in next-generation cancer therapeutics and underscores the need for well-designed clinical studies to facilitate their successful integration into oncology practice.

## 1. Introduction

Cancer remains one of the leading causes of morbidity and mortality worldwide despite substantial advances in early diagnosis and therapeutic interventions. According to the Global Cancer Observatory (GLOBOCAN 2022), approximately 19.9 million new cancer cases and 9.7 million cancer-related deaths were reported globally [1]. Current treatment modalities, including chemotherapy, radiotherapy, immunotherapy, and targeted therapies, have significantly improved patient outcomes; however, their clinical utility is frequently limited by systemic toxicity, multidrug resistance, nonspecific targeting, and tumor recurrence. Consequently, there is an increasing demand for safer and more effective therapeutic strategies for cancer prevention and treatment.

**Figure 1:**
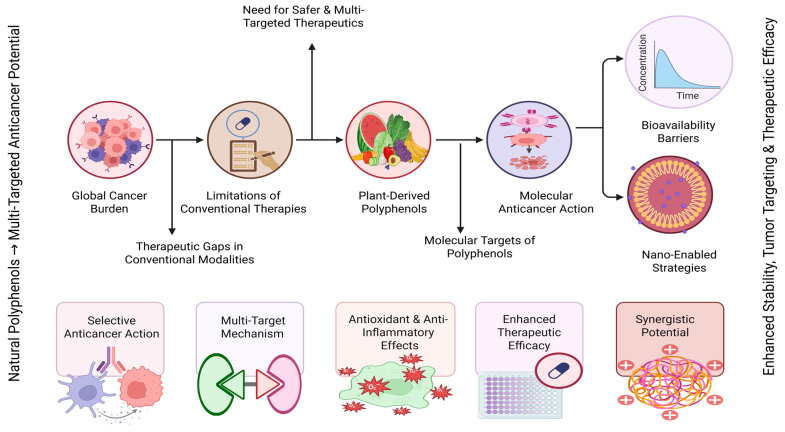
Benefits of Anticancer activity and bioavailability of Plant-Derived Polyphenols.

In this context, plant-derived bioactive compounds have attracted considerable attention as potential alternatives or adjuncts to conventional anticancer agents due to their ability to modulate multiple molecular pathways involved in tumor initiation and progression, including apoptosis, oxidative stress, inflammation, angiogenesis, and metastasis. In addition, polyphenols generally exhibit low systemic toxicity and may enhance the therapeutic efficacy of conventional chemotherapy and radiotherapy while reducing treatment-associated adverse effects. The multifaceted anticancer activities and bioavailability-associated advantages of plant-derived polyphenols are summarized in Figure 1. Among these, polyphenols represent a diverse group of naturally occurring secondary metabolites abundantly present in fruits, vegetables, cereals, tea, wine, and medicinal plants [2]. Epidemiological and experimental studies suggest that dietary habits substantially influence cancer risk, with the American Institute for Cancer Research estimating that nearly 30–40% of cancers may be preventable through appropriate dietary and lifestyle modifications [3,4]. In recent years, polyphenols have emerged as promising anticancer agents due to their broad spectrum of pharmacological activities, including antioxidant, anti-inflammatory, antiproliferative, pro-apoptotic, cardioprotective, and neuroprotective effects [5].

At the molecular level, polyphenols regulate multiple signaling pathways involved in cancer initiation and progression, including PI3K/Akt/mTOR, MAPKs, NF-κB, STAT3, and Wnt/β-catenin pathways [6]. Through modulation of these signaling cascades, polyphenols can inhibit tumor cell proliferation, angiogenesis, metastasis, and inflammation while promoting apoptosis and cell-cycle arrest. Furthermore, growing evidence indicates that polyphenols can modulate the tumor microenvironment, oxidative stress, immune responses, and epigenetic mechanisms, thereby contributing to their multifaceted anticancer effects.

Despite these promising therapeutic properties, the clinical translation of polyphenols remains challenging because of their poor aqueous solubility, limited intestinal permeability, rapid metabolism, chemical instability, and low systemic bioavailability. These limitations often result in inadequate drug concentrations at the tumor site, thereby reducing therapeutic efficacy. To overcome these barriers, Davatgaran-Taghipour et al. studied the development of nanoformulation of natural polyphenols, including nanoparticles, nano suspensions, liposomes, polymeric nanoparticles, and gold nanoparticles, which have demonstrated remarkable anticancer potential [7].

Several recent review articles, such as Lyubitelev et al., 2023 [6] and Ahmed et al., 2025 [8], have summarized the anticancer potential of dietary polyphenols, particularly focusing on their antioxidant activity, signaling pathway modulation, and chemopreventive effects. For instance, previous reviews on polyphenols as anticancer agents have mainly discussed apoptosis induction, oxidative stress regulation, and inhibition of tumor progression through pathways such as PI3K/Akt, MAPKs, and NF-κB [6,8]. Other reports have specifically focused on individual subclasses of polyphenols, including flavonoids, stilbenes, and phenolic acids, or on specific cancer types such as breast, colorectal, and prostate cancer [7]. In addition, several studies, like Davatgaran-Taghipour et al., 2017 and Jia et al., 2023, have highlighted nanotechnology-based delivery systems designed to improve the bioavailability and therapeutic efficacy of selected polyphenols [7,9]. However, many of these reviews remain limited to specific compounds, cancer models, or isolated delivery approaches and do not comprehensively address the interconnected challenges of bioavailability, targeted delivery, and translational applicability. Moreover, emerging delivery platforms such as polymeric nanocarriers, stimuli-responsive formulations, and multifunctional targeted delivery systems have not been systematically integrated within a single comprehensive framework. Therefore, the present review differs from previous reports by providing an integrated overview of polyphenol classification, molecular anticancer mechanisms, bioavailability-associated limitations, and recent advances in targeted delivery strategies, while also emphasizing their translational and clinical relevance in precision cancer therapy.

The present review aims to provide a comprehensive overview of the role of polyphenols in cancer management, with particular emphasis on their classification, molecular mechanisms of anticancer action, and current clinical relevance. Furthermore, this review critically discusses the major factors affecting the bioavailability of polyphenols and highlights recent advances in delivery strategies, including nanoformulations, polymeric carriers, hydrogels, and targeted drug delivery systems, designed to improve their stability, pharmacokinetic behavior, and therapeutic efficacy. Collectively, this review seeks to provide an integrated perspective on the translational potential of polyphenol-based therapeutics and their future prospects in cancer therapy and precision medicine.

## 2. Methodology

To prepare this review on plant-derived polyphenols in cancer therapy, the authors performed a systematic literature search and included peer-reviewed studies related to the classification, molecular mechanisms, anticancer activity, bioavailability, pharmacokinetic limitations, and advanced delivery strategies of polyphenols. The literature search was primarily restricted to studies published between 2000 and 2025 to identify MeSH terms, keyword variations, and terminology associated with plant-derived polyphenols and cancer therapeutics. A comprehensive list of search terms was compiled including flavonoids, phenolic acids, stilbenes, lignans, quercetin, resveratrol, genistein, apigenin, gallic acid, caffeic acid, apoptosis, oxidative stress, inflammation, angiogenesis, metastasis, PI3K/Akt, MAPK, NF-κB, bioavailability, pharmacokinetics, gut microbiota, nanoparticles, liposomes, nanoemulsions, phytosomes, polymeric nanocarriers, and targeted drug delivery systems. Earlier studies were selectively included when they provided foundational insights into polyphenol chemistry, anticancer mechanisms, or therapeutic applications. Databases searched included PubMed, Scopus, Web of Science, and Embase. Studies unrelated to cancer, non-English publications, conference abstracts without full text, duplicate records, and articles lacking sufficient experimental evidence were excluded. In addition to database searches, the reference lists of selected articles were manually screened to identify additional relevant studies not captured during the initial search.

Furthermore, for the tabular summary of the anticancer activity of polyphenols, research articles were selected based on their relevance to the evaluation of polyphenols across various cancer models. Inclusion was restricted to peer-reviewed studies providing clear experimental evidence supporting the anticancer effects of polyphenols. Preference was given to studies offering detailed mechanistic insights and employing validated experimental models, including human cancer cell lines and animal studies. Articles lacking mechanistic evidence, containing insufficient experimental details, reporting duplicate findings, or focusing on unrelated pharmacological activities were excluded from the tabular summary. For the clinical studies summary, various databases and websites, including ClinicalTrials.gov, were systematically explored. Phase I and Phase II randomized clinical trials investigating resveratrol and other major polyphenols were selected for inclusion. Only completed clinical trials were considered in the tabular summary, whereas ongoing, terminated, suspended, or withdrawn studies were excluded.

## 3. Classification of Polyphenols

Polyphenols are a large and structurally diverse group of naturally occurring secondary metabolites characterized by the presence of one or more aromatic rings bearing multiple hydroxyl groups. These compounds are widely distributed in plants and contribute significantly to plant defense, pigmentation, growth regulation, and protection against environmental stress [10]. Biosynthetically, polyphenols are primarily produced through two major metabolic pathways: the polyketide pathway and the shikimic acid/phenylpropanoid pathway.

In the polyketide pathway, polyphenolic compounds are synthesized through sequential condensation reactions involving activated two-carbon acetate units. These reactions generate polyketide intermediates that subsequently undergo cyclization, reduction, oxidation, and other structural modifications to produce diverse classes of polyphenols [11]. In contrast, the shikimic acid pathway utilizes carbohydrate precursors derived from glycolysis and the pentose phosphate pathway to synthesize aromatic amino acids, including phenylalanine, tyrosine, and tryptophan. Among these, phenylalanine serves as the principal precursor for phenolic biosynthesis through the action of the enzyme phenylalanine ammonia-lyase (PAL), which converts phenylalanine into cinnamic acid. Subsequent enzymatic transformations generate hydroxycinnamic acids such as caffeic acid and ferulic acid, which function as key intermediates in the phenylpropanoid pathway and serve as precursors for the biosynthesis of multiple polyphenolic subclasses [12].

**Figure 2:**
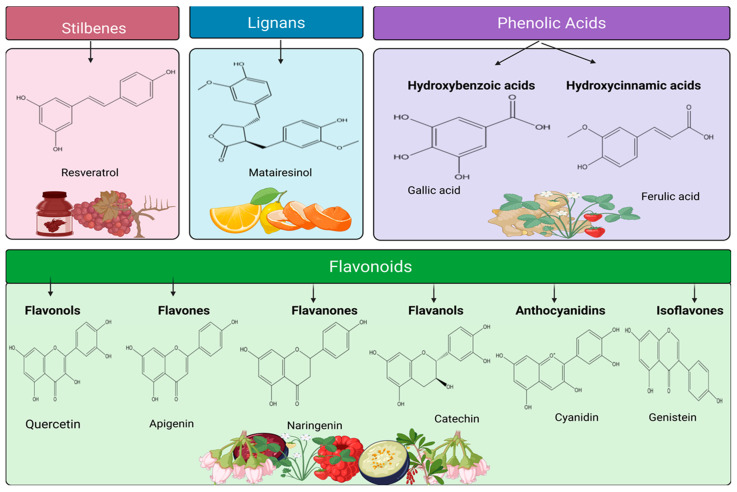
Structural classification of major plant-derived polyphenols with representative chemical scaffolds.

Polyphenols are structurally classified according to the arrangement of aromatic rings, degree of hydroxylation, and linkage patterns within their core skeletons. The major subclasses include flavonoids, phenolic acids, stilbenes, and lignans, each possessing characteristic chemical scaffolds and representative bioactive compounds (Figure 2).

### 3.1. Flavonoids

Flavonoids constitute the largest and most extensively studied class of polyphenols. Structurally, they possess a characteristic C6–C3–C6 skeleton consisting of two aromatic benzene rings (A and B rings) linked through a heterocyclic pyran ring (C ring) containing an oxygen atom, thereby forming a benzo-γ-pyrone framework [13]. In plants, flavonoids commonly occur either as aglycones (sugar-free forms) or as glycosides, in which sugar moieties are attached to the flavonoid backbone.

Based on variations in the oxidation state and substitution pattern of the heterocyclic ring, flavonoids are further categorized into several subclasses, including:

- Flavonols (e.g., quercetin and kaempferol)
- Flavones (e.g., apigenin and luteolin)
- Flavanones (e.g., hesperidin and naringenin)
- Flavanols or catechins (e.g., epigallocatechin gallate)
- Anthocyanidins/anthocyanins (pigmented flavonoids responsible for red and blue coloration in plants)
- Isoflavones (e.g., genistein and daidzein)

Flavonoids exhibit diverse pharmacological activities, including antioxidant, anti-inflammatory, antimicrobial, cardioprotective, neuroprotective, and anticancer effects [13].

### 3.2. Phenolic Acids

Phenolic acids are another major class of polyphenols and are primarily divided into two groups based on their carbon skeletons: hydroxybenzoic acids and hydroxycinnamic acids. Hydroxybenzoic acid derivatives contain a C6–C1 structure and include compounds such as gallic acid, protocatechuic acid, and vanillic acid. Hydroxycinnamic acids possess a C6–C3 structure and include caffeic acid, ferulic acid, sinapic acid, and p-coumaric acid.

These compounds are widely distributed in fruits, vegetables, coffee, cereals, and medicinal plants. Phenolic acids are recognized for their strong antioxidant potential and their ability to modulate inflammatory responses, oxidative stress, and carcinogenic signaling pathways [14].

### 3.3. Stilbenes

Stilbenes are characterized by a C6–C2–C6 skeleton comprising two aromatic rings linked by an ethylene bridge. These compounds are synthesized by plants mainly in response to biotic and abiotic stress conditions such as microbial infection, ultraviolet radiation, oxidation, and heat stress [14]. Among stilbenes, resveratrol is the most extensively investigated compound and is predominantly found in grapes, berries, peanuts, and red wine [15].

Resveratrol has gained significant attention due to its broad spectrum of biological activities, including antioxidant, anti-inflammatory, cardioprotective, neuroprotective, and anticancer effects. Mechanistically, it has been shown to modulate multiple signaling pathways involved in tumor progression, apoptosis, angiogenesis, and metastasis.

### 3.4. Lignans

Lignans are structurally distinct polyphenols formed through the dimerization of two phenylpropanoid (C6–C3) units linked at their β-carbons (C8–C8′ linkage). Their fundamental structure consists of two coniferyl alcohol residues forming a 2,3-dibenzylbutane skeleton [16]. Structural diversity among lignans arises from differences in oxidation patterns and the substitution of methoxy and hydroxyl groups on the aromatic rings.

Lignans are commonly found in flaxseeds, sesame seeds, whole grains, legumes, fruits, and vegetables. Following ingestion, plant lignans are metabolized by intestinal microbiota into mammalian lignans such as enterodiol and enterolactone, which exhibit phytoestrogenic and anticancer properties. Increasing evidence suggests that lignans may contribute to the prevention of hormone-dependent cancers through modulation of estrogen signaling, oxidative stress, and inflammatory pathways [16].

## 4. Molecular Basis of Anticancer Activity

**Figure 3:**
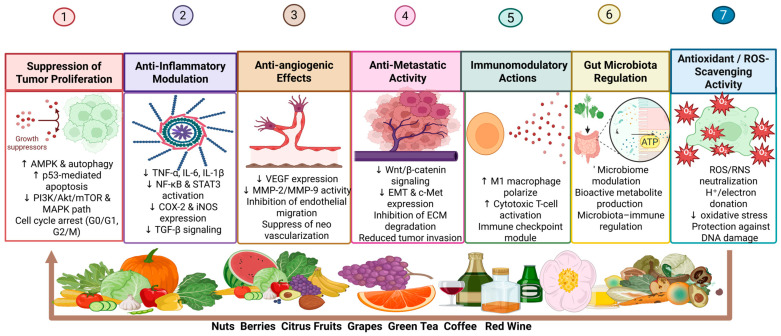
Multifaceted molecular and microbiota-mediated mechanisms underlying the anticancer activity of plant-derived polyphenols: (1) Suppression of tumor proliferation through modulation of AMPK, p53, PI3K/Akt/mTOR, and MAPK signaling pathways. (2) Anti-inflammatory effects mediated through inhibition of TNF-α, IL-6, IL-1β, NF-κB, STAT3, COX-2, and TGF-β signaling. (3) Anti-angiogenic activity via suppression of VEGF expression, endothelial migration, and neovascularization. (4) Anti-metastatic effects through inhibition of Wnt/β-catenin signaling, epithelial–mesenchymal transition (EMT), c-Met expression, and extracellular matrix degradation. (5) Immunomodulatory actions involving macrophage polarization, cytotoxic T-cell activation, and immune checkpoint regulation. (6) Gut microbiota-mediated regulation through microbial biotransformation and production of bioactive metabolites. (7) Antioxidant and reactive oxygen species (ROS)-scavenging activity contributing to protection against oxidative stress and DNA damage.

Polyphenols exert anticancer activity through modulation of multiple molecular and cellular signaling pathways involved in tumor initiation, progression, and metastasis. The major molecular, immunological, anti-metastatic, anti-angiogenic, and microbiota-mediated mechanisms involved in the anticancer activity of polyphenols are summarized in Figure 3. These bioactive compounds regulate oxidative stress by scavenging reactive oxygen species (ROS) and enhancing endogenous antioxidant defense mechanisms, while also modulating redox-sensitive pathways, such as NF-κB, Nrf2, PI3K/Akt, and MAPK signaling. Despite their structural diversity, major polyphenols exert anticancer effects through the modulation of several overlapping signaling pathways and cellular processes, as summarized in Table 1.

### 4.1. Modulation of Oxidative Stress and Cellular Redox Signaling

Oxidative stress plays a critical role in cancer initiation, progression, and therapeutic resistance through excessive production of reactive oxygen species (ROS), which can induce oxidative damage to proteins, lipids, and DNA, ultimately triggering apoptosis or necrosis [17]. Polyphenols exert anticancer effects by regulating cellular redox homeostasis and modulating oxidative stress-mediated signaling pathways. Compounds such as curcumin, resveratrol, quercetin, and epigallocatechin gallate (EGCG) possess strong antioxidant activity due to their ability to scavenge free radicals and enhance endogenous antioxidant defense systems through activation of the Nrf2 pathway. This leads to increased expression of antioxidant enzymes including superoxide dismutase (SOD), catalase, and glutathione peroxidase (GPx), thereby reducing oxidative damage and inflammation-associated carcinogenesis. The mechanism of action of polyphenols as antioxidant, anti-proliferative, anti-inflammatory, anti-angiogenic and anti-metastatic agents is depicted in Figure 2.

Under tumor-specific conditions, polyphenols may also exhibit pro-oxidant effects by increasing intracellular ROS levels beyond the tolerance threshold of cancer cells, resulting in mitochondrial dysfunction, cytochrome c release, and caspase-mediated apoptosis. Curcumin and resveratrol, for instance, have been shown to induce ROS-mediated apoptosis in breast, colon, and lung cancer cells by disrupting mitochondrial electron transport and impairing redox balance [18].

Polyphenols such as curcumin, quercetin, and resveratrol further suppress redox-sensitive oncogenic pathways such as NF-κB, PI3K/Akt, MAPK, and JAK/STAT signaling, thereby inhibiting tumor cell proliferation, inflammation, angiogenesis, and metastasis. Additionally, compounds like resveratrol and EGCG downregulate hypoxia-inducible factor-1α (HIF-1α) and vascular endothelial growth factor (VEGF), contributing to antiangiogenic effects. Through these combined antioxidant, pro-oxidant, and signaling-modulatory actions, polyphenols play a significant role in cancer prevention and therapy.

### 4.2. Regulation of Programmed Cell Death

#### 4.2.1. Polyphenol-Mediated Apoptosis

Apoptosis or programmed cell death, is a crucial mechanism for eliminating damaged and malignant cells, and its dysregulation contributes significantly to cancer progression, metastasis and therapeutic resistance [19,20]. Polyphenols exert anticancer effects by activating both intrinsic (mitochondrial-mediated) and extrinsic (death receptor-mediated) apoptotic pathways [21]. Compounds such as curcumin, resveratrol, quercetin, apigenin, epigallocatechin gallate (EGCG), and genistein regulate the expression of pro- and anti-apoptotic proteins by increasing the Bax/Bcl-2 ratio, promoting mitochondrial membrane depolarization, cytochrome c release, and activation of caspases including caspase-3 and caspase-9. Curcumin and resveratrol have also been shown to activate the tumor suppressor protein p53, resulting in cell cycle arrest and induction of apoptosis in various cancer models.

Polyphenols also inhibit oncogenic survival pathways such as NF-κB, PI3K/Akt, STAT3, and MAPK, thereby suppressing anti-apoptotic proteins including Bcl-2, survivin, and XIAP. Certain polyphenols, particularly EGCG and quercetin, additionally induce ROS-mediated mitochondrial dysfunction, leading to selective apoptosis in cancer cells. In the extrinsic pathway, polyphenols enhance Fas/FasL and TRAIL-mediated signaling, resulting in activation of caspase-8 and apoptotic cell death. Through these multiple mechanisms, polyphenols effectively promote apoptosis and inhibit tumor progression. Naringenin induced apoptosis in gastric cancer cells (SGC-7901) by increasing the expression of caspase-3, p53, and Bax while simultaneously downregulating the anti-apoptotic proteins Bcl-2 and survivin [22,23]. Similarly, hesperetin promoted apoptosis in prostate cancer cells through inhibition of the NF-κB signaling pathway and suppression of Bcl-2 transcription and translation [24]. Daidzein also exhibited pro-apoptotic activity in the SK-Hep-1 cell line by upregulating BAK expression and reducing the levels of multiple anti-apoptotic proteins [25].

#### 4.2.2. Polyphenol-Induced Autophagy

Autophagy is a regulated intracellular degradation process that maintains cellular homeostasis by removing damaged organelles and proteins [26]. In cancer, autophagy plays a dual role, acting as both a tumor-suppressive mechanism and a survival strategy under metabolic stress. Polyphenols modulate autophagy and contribute to anticancer activity through regulation of multiple signaling pathways [27].

Several polyphenols, including resveratrol, curcumin, epigallocatechin gallate (EGCG), quercetin, and fisetin, induce autophagy primarily through inhibition of the PI3K/Akt/mTOR pathway and activation of AMP-activated protein kinase (AMPK). These effects promote autophagosome formation and increase expression of autophagy-related proteins such as Beclin-1, LC3-II, and Atg proteins [28,29]. Curcumin and resveratrol have been shown to induce autophagic cell death in various cancer models, while EGCG and quercetin regulate ROS-mediated autophagic signaling [17]. Kaempferol exerts therapeutic effects by modulating autophagy and endoplasmic reticulum stress pathways, thereby protecting against cancer [30].

Polyphenols also influence the crosstalk between autophagy and apoptosis, thereby sensitizing cancer cells to programmed cell death and improving therapeutic responses. Furthermore, modulation of protective autophagy by polyphenols may help overcome chemoresistance and enhance the efficacy of conventional anticancer therapies [28].

### 4.3. Inhibition of Cell Proliferation and Cell Cycle Progression

Uncontrolled cell proliferation and dysregulated cell cycle progression are major hallmarks of cancer. Polyphenols exert anticancer effects by modulating key regulators of cell cycle control and proliferative signaling pathways. Compounds such as curcumin, resveratrol, quercetin, genistein, apigenin, and epigallocatechin gallate (EGCG) induce cell cycle arrest at G0/G1, S, or G2/M phases, thereby suppressing tumor cell growth [30].

Polyphenols regulate the expression of cyclins, cyclin-dependent kinases (CDKs), and CDK inhibitors. Curcumin and quercetin suppress cyclin D1, cyclin E, CDK2, and CDK4 expression, thereby preventing the transition from G1 to S phase. Resveratrol and genistein enhance expression of p21, p27, and p53, leading to inhibition of cell cycle progression. In addition, polyphenols suppress oncogenic pathways including PI3K/Akt, MAPK, STAT3, Wnt/β-catenin, and EGFR signaling, thereby reducing cancer cell proliferation and survival. Through these multitargeted mechanisms, polyphenols effectively inhibit tumor growth and may improve responses to anticancer therapy [31].

### 4.4. Suppression of Angiogenesis and Metastasis

Angiogenesis and metastasis are essential processes in tumor progression and cancer dissemination. Polyphenols exhibit anticancer effects by targeting multiple molecular pathways involved in these events. Compounds such as resveratrol, curcumin, epigallocatechin gallate (EGCG), quercetin, luteolin, and apigenin suppress angiogenesis through inhibition of vascular endothelial growth factor (VEGF), hypoxia-inducible factor-1α (HIF-1α), and signaling pathways including PI3K/Akt and MAPK. These effects reduce endothelial cell proliferation and the formation of new blood vessels within tumors.

Polyphenols also inhibit cancer cell migration and invasion by suppressing matrix metalloproteinases (MMP-2 and MMP-9) and regulating epithelial-to-mesenchymal transition (EMT)-associated proteins such as E-cadherin and vimentin. In addition, they modulate inflammatory pathways, including NF-κB and STAT3, which contribute to metastatic progression. Through these combined mechanisms, polyphenols effectively limit tumor growth, invasion, and metastasis [32,33].

### 4.5. Modulation of Inflammatory and Immune Signaling Pathways

Chronic inflammation contributes significantly to cancer in initiation, progression, metastasis, and therapeutic resistance [34,36,38]. Polyphenols exert anticancer effects by modulating inflammatory mediators and immune signaling pathways within the tumor microenvironment. Compounds such as curcumin, resveratrol, quercetin, epigallocatechin gallate (EGCG), apigenin, and anthocyanins suppress pro-inflammatory cytokines including tumor necrosis factor-α (TNF-α), interleukin-6 (IL-6), and interleukin-1β (IL-1β) through inhibition of nuclear factor-kappa B (NF-κB), cyclooxygenase-2 (COX-2), signal transducer and activator of transcription 3 (STAT3), and inducible nitric oxide synthase (iNOS) signaling pathways. These effects reduce inflammation-associated tumor growth and survival [34,35].

Polyphenols also regulate immune responses by modulating macrophages, dendritic cells, natural killer (NK) cells, and T lymphocytes. EGCG and quercetin have been shown to enhance antitumor immunity by improving cytotoxic T-cell activity and reducing immunosuppressive signaling within the tumor microenvironment. Additionally, certain polyphenols may influence immune checkpoint pathways and decrease tumor immune evasion. Through these combined anti-inflammatory and immunomodulatory actions, polyphenols contribute significantly to cancer prevention and therapy [39].

### 4.6. Epigenetic Regulation

Epigenetic alterations, including DNA methylation, histone modifications, and dysregulation of non-coding RNAs, play a critical role in cancer initiation and progression by influencing gene expression without altering the DNA sequence. Polyphenols have emerged as important epigenetic modulators capable of reversing abnormal epigenetic changes associated with tumor development.

Several polyphenols, including curcumin, resveratrol, epigallocatechin gallate (EGCG), genistein, and quercetin, regulate the activity of DNA methyltransferases (DNMTs), histone deacetylases (HDACs), and histone acetyltransferases (HATs), thereby restoring the expression of tumor suppressor genes and inhibiting oncogenic signaling [36]. EGCG and genistein have been shown to reduce aberrant DNA methylation, while curcumin and resveratrol modulate histone acetylation and chromatin remodeling. Moreover, polyphenols regulate oncogenic and tumor suppressor microRNAs, including miR-21, miR-34a, and miR-200 family members, thereby influencing proliferation, invasion, and metastasis [37].

Through modulation of epigenetic mechanisms, polyphenols suppress tumor growth, enhance apoptosis, and improve sensitivity to anticancer therapies, highlighting their potential as promising agents in cancer prevention and treatment.

## 5. Anticancer Effect of Polyphenols

The polyphenols have shown anticancer activities against different cancers via different mechanisms. The most commonly used polyphenols against different cancers have been described in the following section and illustrated in Table 2, Table 3, Table 4 and Table 5.

### 5.1. Phenolic Acids

Phenolic acids possess significant cytotoxic potential primarily due to their strong antioxidant properties, including free radical scavenging activity, enhancement of intracellular glutathione (GSH) levels, metal-chelating ability, and regulation of transcription factors such as nuclear factor erythroid 2–related factor 2 (NRF2) [17]. These mechanisms collectively help reduce oxidative stress, modulate cellular redox balance, inhibit DNA damage, and suppress cancer cell proliferation and survival [31]. Additionally, they prevent proliferation of cells via extracellular signal regulated kinase (Erk)1/2, cyclin dependent kinases (CDKs), angiogenic factors such as Vascular Endothelial Growth Factor (VEGF), and also prevent migration and metastasis [18]. The cytotoxic potential of some phenolic acids is described in a further section.

Vanillic acid, a naturally occurring phenolic acid, exhibits significant anticancer activity against multiple cancer types through modulation of diverse molecular pathways. It suppresses tumor proliferation by inhibiting HIF-1α and related mTOR and ERK signaling pathways in colon cancer cells [40]. In endometrial cancer models, vanillic acid modulates oxidative stress markers, antioxidant defense systems, and matrix metalloproteinases, thereby influencing tumor progression and carcinogenesis [41]. It also induces apoptosis by upregulating caspase-3, Bad, Nrf-2, and GSTA-5 while downregulating cyclin D1 and Bcl-2 expression [42]. Studies have further demonstrated its ability to inhibit lung cancer cell proliferation and stimulate antitumor immune responses through STING pathway activation in breast cancer [43,44]. Additionally, vanillic acid reduced lipid peroxidation and restored antioxidant defense systems in oral cancer models [45]. Overall, vanillic acid demonstrates antioxidant, anti-inflammatory, pro-apoptotic, and immunomodulatory activities, highlighting its promise as a potential therapeutic phytochemical in cancer management.

Gallic acid selectively inhibited the growth and angiogenesis in ovarian cancer cell lines OVCAR-3 and A2780/CP70 in a concentration-dependent manner via inhibition of VEGF secretion through suppression of Akt phosphorylation and HIF-1α expression and promotion of PTEN expression [46].

In cervical cancer cells HeLa and human umbilical vein endothelial cells (HUVEC), gallic acids led to apoptotic cell death via the induction of ROS and GSH, accompanied by the loss of mitochondrial membrane potential [47]. In glioblastoma multiforme (T98G cells), gallic acid exhibited anticancer activity by modulating the expression of miR-17-3p, p21-associated miR-21-5p, and ATM-related miR-421-5p, thereby influencing cell cycle regulation and DNA damage response pathways [48]. In prostate cancer cell PC3, it reduced proliferation and invasion via diminishing pSTAT3, pERK1/2, and pAKT signaling proteins [49]. Sanchez-Martin et al. reported that Gallic acid exhibited antitumor efficacy via involvement with DNA G-quadruplexes (G4s) in colorectal cancer [50].

Similarly, caffeic acid and Caffeic acid Phenyl ester possess broad-spectrum anticancer activities across melanoma [51,52], breast [53], prostate [54], lung [55], oral [56], ovarian [57], and nasopharyngeal cancers [58]. Their mechanisms involve induction of apoptosis, inhibition of proliferation, suppression of migration and invasion, and regulation of major oncogenic signaling pathways such as Akt, NF-κB, and cell cycle regulatory proteins. The anticancer potential of phenolic acids is summarized in Table 2.

**Table 2:** Anticancer potential of Phenolic acids in different cancers.

Polyphenol	Type of Cancer	Experimental Model/Cell Line	Mechanism of Action	Reference
Vanillic acid	Colon	HCT116 colon cancer cell line	Inhibition of HIF-1α expression by suppressing mammalian target of rapamycin/p70 ribosomal protein S6 kinase/eukaryotic initiation factor 4E-binding protein-1 and Raf/extracellular signal-regulated kinase (ERK) kinase (MEK)/ERK pathways.	[40]
	Endometrial	Albino female (185–215 g) Wistar rats	Modulation of oxidative stress markers, antioxidant defense systems, and matrix metalloproteinases, upregulated expression of MMP-2 and 9 and cyclin D1	[41]
	Liver and colon	HepG2 cell line	Induction of the expression of GSTA-5 and Nrf-2 genes; reduction in Cyclin D1; Up-regulation of Caspases-3 and Bad levels; Down-regulation of the Bcl-2 level.	[42]
	Lung	Lung cancer cell line	Attenuation of cell proliferation, xenobiotic enzyme activity and pulmonary mitochondrial enzyme alterations	[43]
	Breast	Balb/c Mice, SKBR3 cell line	STING activation in macrophages leading to antineoplastic activity	[44]
	Oral	Oral cancer hamster	Reduction in lipid peroxidation and improved antioxidant status	[45]
Gallic acid	Ovarian	OVCAR-3 and A2780/CP70,	Downregulation of AKT phosphorylation, HIF-1α expression Promotion of PTEN expression	[46]
	Cervical	HeLa cells human umbilical vein endothelial cells (HUVEC)	Induction of ROS and GSH accompanied by the loss of mitochondrial membrane potential	[47]
	Glioblastoma multiforme	T98G cell line	Alteration in expression of (mir-17-3p), p-21 protein (mir-21-5p) and ATM (mir-421-5p)	[48]
	Prostate	PC3 cell	IL-6 down-regulation and decreased IL-6 protein level	[49]
	Colorectal	CRL1790, SW480 and SW620	Interaction with DNA G-quadruplexes	[50]
Caffeic acid	Melanoma	SK-Mel-28 cell line	Reduction in cell viability and induction of apoptosis	[51]
	Melanoma	B16 melanoma cells	Inhibition of melanin synthesis through different biochemical mechanisms	[52]
	Breast	MCF-7 cell line	Decrease in cell viability, Cell death induction by apoptosis, inhibition of colony formation, modulation of the cell cycle and alterations in gene expression of caspases	[53]
	Prostate	Androgen-independent prostate cancer cells	Cell cycle arrest and growth inhibition via regulation of Skp2, p53, p21Cip1 and p27Kip1	[54]
	Lung	Lung adenocarcinoma cells	Suppression of motility promoted by TGF-β through Akt inhibition	[55]
	Oral	Oral cancer cells	Anticancer activity through apoptosis induction and inhibition of tumor progression	[56]
Caffeic acid phenyl ester	Ovarian	SKOV3 cells	Suppression of nuclear factor kappa b (NF-κB) through the inhibition of IκB phosphorylation, nuclear translocation of p65 and NF-κB p65 DNA binding activity.	[57]
	Nasopharyngeal	TW01, TW04 cells	Upregulation of NDRG1 expression via the MAPK pathway and by inhibiting phosphorylation of STAT3	[58]

The above studies suggest that phenolic acids exert anticancer effects mainly by modulating oxidative stress, inflammation, apoptosis, and cell-cycle regulation. While generally less cytotoxic than some flavonoids, they consistently influence the tumor microenvironment and inhibit pathways involved in cancer progression. A key trend emerging from the literature is their ability to restore redox balance and suppress chronic inflammation, both major drivers of tumor development. These findings highlight the potential of phenolic acids as complementary agents for cancer prevention and management.

### 5.2. Flavonoids

Flavonoids exhibited anticancer efficacy due to their antioxidant activity, apoptotic mechanism via reducing bcl-2 and bcl-xL and increasing the levels of p-53 gene, caspase-3, and inhibition of NF-κB signaling up-regulation of BAK and down-regulation of various anti-apoptotic proteins. Flavonoids show anticancer properties by impairing various signaling cascades like MAPK, PI3K/Akt/mTOR, Wnt/β-catenin and AMPK. In addition, they play a role in the complex process of metastatic spread, such as MMPs, uPA/uPAR, TGF-β, and other regulators of the epithelial–mesenchymal transition [59]. They exhibited cytotoxic potential via different signaling pathways, such as the NF-κB pathway [60,61].

Souza et al., 2017 [62], studied the effect of Apigenin on different cervical cancer cell lines, HeLa (human papillomavirus/HPV 18-positive), SiHa (HPV 16-positive), CaSki (HPV 16 and HPV 18-positive), and C33A (HPV-negative) and the human epithelial cell line (HaCaT). The results showed that apigenin had a selective cytotoxic effect and induced apoptosis in cancerous cells via inducing mitochondrial impairment [62]. Apigenin has also shown anticancer potential in colorectal [63], breast [64], lung [65], and prostate cancers [66].

Tangeretin inhibited breast cancer cell proliferation through induction of CYP1A1 and CYP1B1 enzymes, leading to formation of the active metabolite 4′-hydroxy tangeretin, which contributed to anticancer activity [67]. Tangeretin suppressed lung cancer progression in mice by modulating NF-κB/ICAM-1 and JAK/STAT-3 signaling pathways while simultaneously promoting apoptosis [68]. Tangeretin also showed the anticancer efficacy against colorectal cancer [69], Liver cancer [70] and gastric cancer [71].

Genistein promoted apoptosis in laryngeal cancer cells through activation of p53-responsive microRNA-1469, which targeted the anti-apoptotic protein Mcl1 [72]. Genistein effectively inhibited proliferation and induced apoptosis in HT29 colon cancer cells [73] and showed anticancer efficacy in colorectal cancer [74], breast cancer [75], and cervical cancer [76].

Quercetin exhibited its anticancer potential against different cancers [77] via different mechanisms such as reduction in intracellular ROS [78], decreasing level of anti-apoptotic protein level of Mcl-1, Bcl-2, Bcl-x, cyclin-D and increasing level of Bad, Bax, Bid, increasing gene expression of TNFRSF10D, TP53INP1 [79]. Quercetin has also shown anticancer potential in breast cancer [80], Glioblastoma [81], Colon Cancer [82], and oral squamous cell cancer [83]. The anticancer potential of other flavonoids is illustrated in Table 3.

**Table 3:** Anticancer potential of flavonoids in different Cancers.

Polyphenol Category	Polyphenol	Type of Cancer	Experimental Model	Mechanism of Action	Reference
Flavones	Apigenin	Cervical	HeLa, SiHa, CaSki, and C33A cell lines	Selective cytotoxic effect on cancerous cells via inducing mitochondrial impairment	[62]
		Colorectal	DLD1 and SW480 cells	Inhibition of proliferation, invasion and migration via reduced phosphorylation of FAK, Akt	[63]
		Breast	MDA-MB-231 cell line	Suppression of cyclin A, cyclin B, and CDK1; Inhibition of HDAC activity and Induction of histone H3 acetylation	[64]
		Lung	A549 cells	Reduction in the PI3K/Akt signaling pathway	[65]
		Prostate	PC3	Upregulation of p21 and p27 expression along with activation of caspase-8, caspase-3, and TNF-α, and downregulation of PI3K/Akt/NF-κB signaling.	[66]
	Tangeretin	Breast	MCF-7, MDA-MB-468 cells	Induction of CYP1 enzyme activity and CYP1A1/CYP1B1 protein expression	[67]
		Lung	BALB/c mice	Reduction in the expression of NF-κB/ICAM-1 and JAK/STAT, and promoted caspase-3 signal transduction	[68]
		Colorectal	HCT116 Cells	Induction of GADD45α expression and antiproliferative activity	[69]
		Liver	HepG2 cells	Induction of endoplasmic reticulum-mediated autophagy in human hepatoma cells	[70]
		Gastric	AGS, BGC-823, and SGC-7901 cells BALB/c nude mice (5–6 weeks of age)	Up-regulation of RARβ-induced apoptosis	[71]
Isoflavones	Genistein	Laryngeal	TU212 and Hep-2 cell lines	Induction of apoptosis by decreasing Mcl1 expression	[72]
		Colon	HT29 cell line	Reduction in p38 MAPK gene expression and MMP-2 levels along with activation of the caspase-3 pathway.	[73]
		Colorectal	HCT-116, LoVo	Inhibition of Akt phosphorylation	[74]
		Breast	MCF-7, T47D	Increase in pro-inflammatory and reduction in anti-inflammatory gene expression	[75]
		Cervical	HeLa	Reduction in the activity of DNMTs, HDACs, and HMTs and reduced global DNA methylation levels.	[76]
Flavonols	Quercetin	Hepatocellular carcinoma	HepG2 cells	Reduction in intracellular ROS (independent of p53 expression)	[78]
		Gastric	AGS Cells	Down-regulation of proteins (Mcl-1, Bcl-2 and Bcl-x) up-regulation of proteins (Bad, Bax, Bid)	[79]
		Breast	MCF-7, MDA-MB-231	Down-regulation of CyclinD1, p21	[80]
		Glioblastoma	U251	Disruption of the regulation of apoptosis genes such as Bax and Bcl-2, down-regulation of matrix metallopeptidases, like MMP9 and MMP2.	[81]
		Colon	Caco-2	Reduction in MMP-2, MMP-9, TNF-α, COX-2, and IL-6 expression, thereby suppressing inflammation and metastasis	[82]
		Oral squamous cell carcinoma	OSC20, SAS, and HN22 cells	Suppression of cell migration through EMT and matrix metalloproteinase (MMP) in OSCC cells	[83]
	Kaempferol	Colorectal	HCT116, HCT15, and SW480	Induction of PARP cleavage and activation of caspase-8, caspase-9, caspase-3, and phospho-p38 MAPK signaling	[84]
		Bladder	EJ cells and normal bladder cells SV-HUC-1	Inhibition of the function of phosphorylated AKT (p-AKT), CyclinD1, CDK4, Bid, Mcl-1 and Bcl-xL, and promoting p-BRCA1, p-ATM, p53, p21, p38, Bax and Bid expression	[85]
		Cervical	HeLa	Down-regulation of the PI3K/AKT and hTERT pathways	[86]
		Gastric	AGS, SNU-216, NCI-N87, SNU-638, and MKN-74	Activation of the IRE1-JNK-CHOP signaling from cytosol to nucleus, and G9a inhibition, activates autophagic cell death in GC cells	[87]
Flavonones	Hesperetin	Lung	H522	Upregulation of the levels of Fas, FADD, and caspase-8 expression and downregulation of the levels of caspase-3 and caspase-9, p53, and Bax expression	[88]
	Hesperidine	Breast	Rats	Reduction in Ki67 expression	[89]
		Ovarian	A2789 Cells	Induction of apoptosis	[90]
		Hepatocellular	Rats	Inhibition of thioacetamide activated Wnt3α/β-catenin pathways	[91]
	Galangin	Kidney	A498	Upregulation of Bax and cytochrome-c expression along with downregulation of Bcl-2 and inhibition of the PI3K/AKT/mTOR signaling pathway.	[92]
Anthocyanidines	Cyanidin	Glioblastoma	U87 and U251 Cells	Reduction in Skp2, Zeb1, N-cadherin, Increment of Skp2 degradation through the ubiquitin proteasome dependent pathway	[93]
		Nasopharyngeal	NPC-TW039 and NPC-TW 076 cells	Induction of p53-independent S-phase arrest and apoptosis through inhibition of the PI3K-AKT signaling pathway	[94]
		Laryngeal	TU212 and M4e	Suppression of carcinoma progression via modulation of caspase-3 and AKT signaling pathways	[95]
		Retinoblastoma	Y-79, C-33A and WERI-Rb-1	Inhibition of cell progression and induction of apoptosis through activation of PTEN and caspase-3 pathways	[96]
		Liver	MHCC97H	Promotion of apoptosis through suppression of H19 expression	[97]
		Breast	MCF-7	Upregulation of miR-124 expression.	[98]
	Delphinidin	Breast	MDA-MB-453 and BT474 cells	Induction of autophagy via suppression of the mTOR signaling pathway and activation of the AMPK signaling pathway	[99]
		Ovarian	SKOV3	Inactivation of PI3K/AKT and ERK1/2 mitogen-activated protein kinase signaling cascades	[100]
		Prostate	PC3 cells	Suppression of the β-catenin signaling pathway	[101]
		Ovary	SKOV3 ovarian cancer cells	Inhibition of migration and invasion through modulation of BDNF-induced signaling	[102]
		Colorectal	HCT116 cells	Induction of apoptosis via modulation of JAK/STAT3 and MAPK signaling pathways	[103]
		Non-small lung cancer	NSCLC cells	Enhancement of radiotherapeutic effects via autophagy induction and JNK/MAPK pathway activation	[104]

Overall, flavonoids are the most extensively studied class of polyphenols in cancer research and demonstrate a wide range of anticancer activities. Despite their structural diversity, they consistently target key signaling pathways involved in tumor growth, survival, inflammation, angiogenesis, and metastasis. A notable trend across studies is their ability to act on multiple molecular targets simultaneously, particularly through modulation of PI3K/Akt/mTOR, NF-κB, MAPK, and STAT3 pathways. This broad mechanistic activity, together with their generally favorable safety profile, supports their potential use in combination therapies and as adjuvant anticancer agents.

### 5.3. Stilbenes

Within the stilbene class, resveratrol is a naturally occurring phytoalexin synthesized by plants in response to pathogenic attack and environmental stress. Owing to its diverse molecular targets and pleiotropic biological activities, resveratrol has attracted considerable attention as a potential anticancer agent and may represent a promising strategy for advancing cancer therapeutics. Stilbenes are considered to possess significant anticancer properties and have been found to have strong anti-inflammatory and antioxidant properties and can also have a direct killing effect on tumor cells through mechanisms that induce cytotoxicity [105]. By increasing the activity of immunomodulatory T cells and decreasing the levels of pro-inflammatory factors such as MCP-1 and TNF-α, resveratrol can induce cytotoxicity [106,107]. In addition, resveratrol inhibits the proliferation of tumor cells through inhibition of the Wnt/β-catenin signaling pathway, which is an important regulator of the survival and stemness of cancer cells [108,109]. It also exhibited anticancer effect in hepatocellular carcinoma [59], oral squamous cell carcinoma [110], neuroblastoma [111], colorectal [112], cervical cancer [113], leukemia [114], and prostate cancer [115].

Pterostilbene is a naturally occurring dimethylated derivative of resveratrol, predominantly found in blueberries, has also shown strong anticancer activity. It inhibits tumor cell proliferation and initiates apoptosis by downregulating key oncogenic signaling pathways, including the PI3K/Akt, MAPK, and NF-κB pathways. Pterostilbene has shown anticancer potential in small cell lung cancer [116], triple negative breast cancer [117], liver cancer [118], and pancreatic cancer [119]. The anticancer properties of stilbenes are summarized in Table 4.

**Table 4:** Anticancer potential of Stilbene in different cancers.

Polyphenol	Type of Cancer	Experimental Model	Mechanism of Action	Reference
Resveratrol	Oral squamous cell carcinoma	SCC-VII, SCC-25, and YD-38 cells	Induction of cell cycle arrest in the G2/M phase and enhancement in expression of phospho-cdc2 (Tyr 15), cyclin A2, and cyclin B1	[110]
	Malignant melanoma	A375SM cells	Induction of the ROS-p38-p53 pathway and the p53 and ER stress pathway	[105]
	Hepatocellular carcinoma	MHCC97-H	Activation of p53 and inhibition of phosphoinositide 3-kinase/Akt.	[59]
	Neuroendocrine cancer	Mouse neuroblastoma cells Neuro-2a and NB41A3	Induction of ER stress-iROS-involved intrinsic apoptosis Suppression of Rho-dependent cell migration	[111]
	Colorectal cancer	Human HCT116 and SW620	Decreased cell viability, enhanced apoptosis and increased ROS level	[112]
	Leukemia	Human U937 and MOLT-4	Decreased cell viability, DNA fragmentation	[113]
	Cervical	HeLa Cells	Induced mitophagy and ROS overproduction	[114]
	Prostate	Human PC3	Oxidative Phosphorylation	[115]
Pterostilbene	Gallbladder	GBC-SD, SGC-996 and NOZ	Inhibition of PI3K/Akt activation	[109]
	Non-small cell lung cancer	A549 cells	COX-2 suppressed the proliferation	[116]
	Liver	HepG2	Inhibition of cell activity and migration, cell cycle shift	[117]
	TNBC	MDA-MB231	Inhibition of cell proliferation and migration	[118]
	Pancreatic	PDAC	Induction of S-phase, cell cycle arrest, apoptosis and autophagic cell death and inhibiting Multidrug resistance protein 1	[119]

### 5.4. Lignans

Dibenzocyclooctadienes (specifically lignans) have been studied for many different purposes due to their wide variety of biological activities, including potential uses as antivirals, antioxidants, anti-inflammatories, and for treating tumors. Some of these compounds are gomisin (gomisin A/G), schisandrin B and C. Each of these lignans has been shown to have anticancer activity through its actions on various oncogenic signaling pathways. As an example, gomisin is known to have anticancer properties because it inhibits the phosphorylation of Akt and suppresses epithelial–mesenchymal transition (EMT), both of which help reduce the overall proliferation and metastasis of tumor cells. Additionally, gomisin G inhibits the proliferation of MDA-MB-231 and MDA-MB-468 breast cancer cell lines by inhibiting Akt signaling and decreasing the level of phosphorylated retinoblastoma (Rb) protein, leading to cell cycle arrest [120]. Gomicin A suppresses metastatic melanoma progression by inhibiting AMPK signaling and ERK/JNK-mediated survival pathways, leading to reduced proliferation and metastatic potential [121]. Gomisin L1 induces apoptosis in ovarian cancer cells by modulating NADPH oxidase activity, thereby increasing oxidative stress and triggering programmed cell death [122]. Similarly, gomisin J exhibits anticancer effects in breast cancer by inhibiting cancer cell growth and promoting apoptosis via modulation of mitochondrial pathways [123].

In gastric cancer cells, Schisantherin A. induces apoptosis primarily via activation of reactive oxygen species (ROS)-mediated JNK signaling, leading to mitochondrial dysfunction and programmed cell death [124]. In liver cancer models, schisantherin A demonstrates cytotoxic activity supported by both in vitro and in silico analyses [125], while in hepatocellular carcinoma it suppresses tumor growth by disrupting glucose metabolism pathways essential for cancer cell energy production [126]. Additionally, in non-small cell lung cancer, it triggers ferroptosis through activation of the YAP/ACSL4/TfR signaling axis, promoting iron-dependent lipid peroxidation and cell death [127].

Schisantherin B exhibits inhibitory effects on tumor growth and cell viability via Phosphorylation of PI3K/AKT and STA3/JAK2 in prostate cancer [128], while in triple-negative breast cancer, it suppresses progression primarily through inhibition of STAT3 signaling [129]. In colorectal cancer, schisandrin B exerts antitumor activity by modulating the CXCL2/ERK/DUSP11 signaling axis, which leads to reduced proliferation and promotes tumor suppression [130]. Additionally, in colon cancer models, it induces cell cycle arrest and apoptosis, highlighting its ability to disrupt cancer cell division and survival mechanisms [131]. The anticancer effects of lignans are summarized in Table 5.

**Table 5:** Anticancer potential of lignin compounds in different cancers.

Polyphenol	Type of Cancer	Experimental Model	Mechanism of Action	Reference
Gomicin A	Metastatic melanoma	B16F10, A375SM cells	Activation of AMPK, ERK, and JNK and suppression of epithelial–mesenchymal transition (EMT)	[121]
Gomicin L	Ovarian	A2780 and SKOV3 cells	Regulation of intracellular ROS production through NADPH Oxidase (NOX)	[122]
Gomicin G	TNBC	MDA-MB-231 and MDA-MB-468 cells	Inhibition of AKT phosphorylation and reduction in retinoblastoma tumor suppressor protein (Rb) and phosphorylated Rb	[120]
Gomicin J	Breast	MCF-7 and NDA-MB231 Cells	Induction of apoptosis and inhibition of cancer cell proliferation through modulation of mitochondrial apoptotic pathways	[123]
Schisantherin A	Gastric	MKN45 and SGC-7901 cells	ROS-dependent JNK phosphorylation with higher ROS production. Suppression of the Nrf2 factor	[124]
	Liver	HepG2 and Huh7 cells	Induction of apoptosis, ROS generation, and inhibition of cell proliferation	[125]
	Hepatocellular carcinoma	Hep3B and HCCLM3 cells	Regulation of the glucose metabolism pathway leading to inhibition of cell proliferation	[126]
	Non-small cell lung cancer	A549 and H1299 cells	Induction of ferroptosis through activation of the YAP/ACSL4/TfR signaling pathway	[127]
Schisandrin B	Prostate	DU145 and LNCaP cells	Phosphorylation of PI3K/AKT and STA3/JAK2	[128]
	TNBC	MDA-MB-231 and BT-549 cells	Inhibition of the STAT3 signaling pathway leading to suppression of proliferation and metastasis	[129]
	Colorectal	HCT116 and SW480 cells	Regulation of the CXCL2/ERK/DUSP11 signaling pathway causing inhibition of tumor growth	[130]
	Colon	HT-29 and LoVo cells	Induction of cell cycle arrest and apoptosis through mitochondrial-mediated pathways	[131]

The reviewed studies highlight the complementary roles of stilbenes and lignans in cancer management. Stilbenes, particularly resveratrol and its derivatives, exert broad anticancer effects by modulating oxidative stress, cellular metabolism, apoptosis, autophagy, and key oncogenic pathways. Lignans, in contrast, primarily act through antioxidant, anti-inflammatory, and hormone-regulating mechanisms, making them especially relevant in hormone-dependent cancers. Despite their structural and mechanistic differences, both classes target multiple hallmarks of cancer, underscoring the multitarget nature of polyphenols. However, further clinical studies are needed to validate their efficacy and support their therapeutic application.

## 6. Bioavailability of Plant-Derived Polyphenols

The pharmacokinetics and bioavailability of bioactive compounds directly influence their therapeutic efficacy. Several processes, including drug absorption, distribution, metabolism, and elimination, affect the manner in which these compounds exert their effects within the body [132,133]. In the case of polyphenols, factors such as chemical structure, molecular weight, degree of glycosylation, and lipophilicity significantly determine their absorption into the bloodstream. Additionally, the food matrix, chemical stability, aqueous solubility, intestinal permeability, and composition of the gut microbiota also influence the absorption and distribution of polyphenols throughout the body. Collectively, these factors regulate the concentration of polyphenols in systemic circulation and consequently modulate their therapeutic potential [134,135,136]. Factors affecting the bioavailability of polyphenols are discussed below.

### 6.1. Chemical Structure and Food Matrix

The chemical structure of polyphenols has an impact on their solubility, stability and intestinal absorption. Natural sources of flavonoids contain both aglycone and glycosylated forms of flavonoids. Glycosylated flavonoids in most cases will require the activity of β-glucosidases or the gut microbiome to enzymatically hydrolyze the glycoside in these compounds prior to the free form of the polyphenol being available for absorption and therefore potential biological activity [137]. The aglycone form of the flavonoids has better membrane permeability and therefore more rapid absorption than the glycosylated forms, although the degree of absorption of aglycone flavonoids is influenced by the specific type of sugar moiety present and the location of the sugar moiety on the aglycone flavonoid [138].

### 6.2. Poor Aqueous Solubility

Multiple polyphenols have low solubility in water, as shown in Table 6. These compounds have limited permeability through the intestines, thus reducing the ability for the compound to be absorbed into the bloodstream [139]. The impact of low bioavailability on the therapeutic effect is significant.

### 6.3. Chemical and Metabolic Instability

Polyphenolic compounds are generally unstable when exposed to physiological conditions, as they degrade easily due to pH changes, enzymatic hydrolysis, and oxidative reactions. Moreover, metabolites produced from the compounds after ingestion via intestinal or liver metabolism are either not available in sufficient amounts or are in a rapidly transformed state (short half-lives) when they reach the bloodstream; therefore, their therapeutic effect will also be short-lived [144].

### 6.4. Rapid Conjugation and Elimination

Upon absorption into the bloodstream, phase II enzymes (conjugating enzymes) rapidly convert polyphenolic compounds into the following forms: glucuronide, sulfate, and methylated. The conjugated forms of the polyphenolic compounds can be eliminated from the body through either renal or biliary routes. While there may be metabolites of polyphenolic substances that display some biological activity, their anticancer activity usually differs from that of the parent compound (the polyphenol), thereby reducing the overall efficacy of the polyphenolic compound [145].

### 6.5. Influence of Gut Microbiota

Gut microbiota plays a crucial role in the absorption, metabolism, and bioavailability of flavonoids and other polyphenols. Dietary flavonoids can modulate the composition and activity of intestinal microbiota by promoting beneficial bacterial species such as Lactobacillus and Bifidobacterium while suppressing pathogenic microorganisms. In turn, gut microorganisms enzymatically convert complex polyphenols into smaller phenolic acids and other low-molecular-weight metabolites that are more readily absorbed through the intestinal epithelium. Therefore, the health benefits of flavonoids are not only dependent on the parent compounds but also on the metabolites generated through microbial biotransformation. Following ingestion, metabolic conversion mainly occurs within the intestines and liver, where metabolized flavonoid compounds may enhance or reduce bioactivity. Some metabolites may be more bioactive than their parent compounds, potentially contributing to this disparity [146]. These metabolic modifications can either enhance or reduce biological activity and bioavailability. In several cases, microbial metabolites exhibit greater antioxidant, anti-inflammatory, or anticancer activity than the original parent compounds, thereby significantly contributing to the overall therapeutic effects of flavonoids and polyphenols [147].

## 7. Strategies to Enhance Bioavailability and Therapeutic Potential

**Figure 4:**
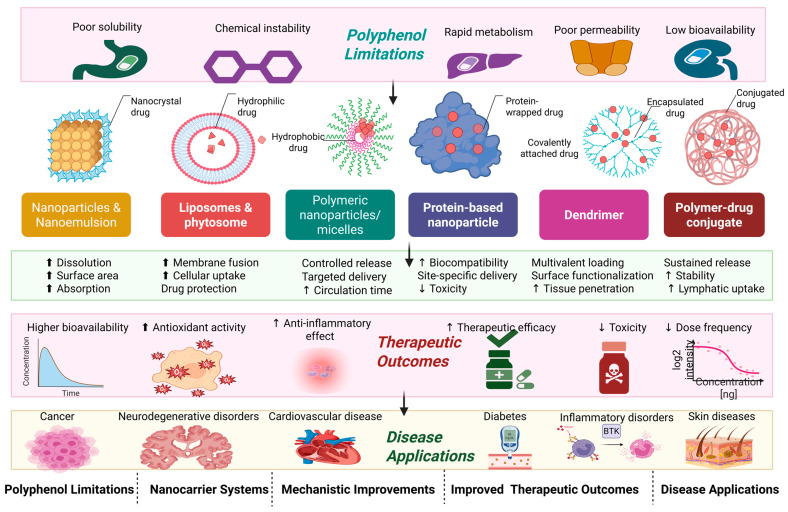
Strategies to enhance the bioavailability and therapeutic potential of Polyphenols.

Although polyphenols have powerful antioxidant, anti-inflammatory, anticancer, and cardioprotective properties, their use in health care is often limited due to several reasons, including low water solubility, instability at room temperature, being easily metabolized, and poor absorption once taken orally. To deal with these challenges, many research groups have developed techniques that will improve polyphenol solubility and stability while providing increased absorption after an oral dose [148]. Nanoparticle system formulations (e.g., solid lipid nanoparticles, nanoemulsions, liposomes, self-microemulsifying drug delivery systems) improve solubility and protect polyphenols from degradation as well as enhance their absorption from the gut. Complexation approaches (cyclodextrin-inclusion complexes, phospholipid complexes, i.e., phytosomes) enhance polyphenol permeability and stability. Chemical modifications (e.g., prodrugs or structural analogs) provide further protection against rapid metabolism and result in improved pharmacokinetic profiles (Figure 4). Bioenhancers like piperine act to inhibit metabolic enzymes, thereby increasing the systemic availability of polyphenols. Table 7 lists a side-by-side comparison of several types of nanocarrier and polymer-based delivery systems that have been developed to improve the bioavailability and therapeutic efficacies of polyphenols.

### 7.1. Nanoparticles

Natural or synthetic polymers can be used to prepare submicron-sized colloidal carriers that are known as polymeric nanoparticles, ranging from 10 to 1000 nm in size. Polymeric nanoparticles are well established as a formulation component for many drug delivery systems. Drug molecules can either be encapsulated in the polymer matrix or adhere to the surface of the polymeric nanoparticles. For this reason, drugs that are encapsulated or adhered to the surface of polymeric nanoparticles are protected from degradation while also improving their stability. Examples of commonly used polymers to develop polymeric nanoparticles include poly(lactic-co-glycolic acid) (PLGA), poly(lactic acid) (PLA), chitosan, alginate, gelatin, and polyethylene glycol (PEG). Polymeric nanoparticles can improve the solubility and bioavailability of poorly soluble drugs while also providing controlled and sustained drug release. Additionally, polymeric nanoparticles can be designed to provide targeted drug delivery through surface modification with ligands and subsequently lessen systemic toxicity [166].

Polymeric nanoparticle platforms have found numerous applications in the development of formulations intended to improve the stability and oral bioavailability of polyphenols. Banik et al. (2022) demonstrated the feasibility of using an emulsion-process method to prepare quercetin-loaded poly(lipoic acid) nanoparticles (QUE/pLA) in an optimized formulation with an average size of ~185 nm [149]. The polymers exhibited an average encapsulation efficiency of 84.8%, providing a sustained release profile, and demonstrated enhanced chemical stability under gastrointestinal pH conditions. Therefore, in vivo studies demonstrated a significantly increased systemic exposure and oral bioavailability of quercetin (29%) when administered as QUE/pLA compared with crystalline quercetin (0.19%). Overall, the results provide evidence that biodegradable polymeric nanoparticles provide an improved option for the oral administration of quercetin. Promising evidence of surface-engineered polymeric nanoparticles has also been found for improving systemic and brain drug delivery of polyphenols. Bagad et al. (2015) developed quercetin-loaded poly(n-butylcyanoacrylate) nanoparticles (QT-PBCA NPs), either with or without a coating of polysorbate-80, to better biodistribute quercetin following oral administration [150]. The spherical nanoparticles created were approximately 161–167 nm in size and had a high entrapment of approximately 75–80%, with their release exhibiting a biphasic profile. The study revealed the presence of both PBCA nanoparticles and polysorbate-80 coating to be effective for enhancing the bioavailability of quercetin. PBCA nanoparticles alone improved the bioavailability of quercetin compared to that in suspension by 2.38 times, while the presence of the polysorbate-80 coating boosted the bioavailability of quercetin by 4.93 times, illustrating how surface modifications can play a significant role in facilitating the intestinal absorption and central nervous system delivery of polyphenols.

Targeted ligand polymer nanoparticles have also recently been studied to enhance the oral absorption and intestinal transport of polyphenols. Siu et al. (2018) [151] utilized galactosylated PLGA nanoparticles loaded with resveratrol (RES-GNPs) produced through solvent diffusion utilizing N-oleoyl-D-galactosamine and Tween 80. The optimized RES-GNPs exhibited an average size of roughly 108 nm (PDI = 0.217), high encapsulation efficiency, and sustained drug release [151]. When given orally, resveratrol contained in the RES-GNPs demonstrated a substantial increase in bioavailability (~335.7%), as compared to the bioavailability attained through oral suspension. The results of the study were verified through in situ intestinal perfusion experiments and cellular uptake studies demonstrating that RES-GNPs have enhanced intestinal permeability and transcellular transport properties, establishing the opportunity to maximize polyphenol absorption through the use of ligand-modified nanoparticles. It is anticipated that bioinspired nanocomposites will also present themselves as viable alternatives to conventional polymeric carriers for increasing the stability of polyphenols. Zhao et al. (2022) [152] have created melanin nanoparticles loaded with epigallocatechin-3-gallate that have a particle size of fewer than 100 nm by incorporating EGCG through π–π stacking and hydrophobic interactions into a polymeric macromolecular structure. The authors report that EGCG@MNPs remains thermally stable and exhibit strong antioxidant activity following high-temperature treatment, and have antimicrobial activity against *E. coli* and *S. aureus* [152].

### 7.2. Liposomes

Liposomes are vesicle-like nanoparticles made up of phospholipid bilayers that have received significant attention regarding the delivery of polyphenols; liposomal structures are amphipathic in nature, allowing for the encapsulation of both hydrophilic and hydrophobic polyphenols (e.g., curcumin, resveratrol, quercetin) within the liposomal lipid bilayer and in the aqueous core, respectively, thereby enhancing solubility and stability. Due to their ability to protect polyphenols through encapsulation from oxidative degradation, enzymatic metabolism, and low gastrointestinal stability, liposomes have the potential to improve the bioavailability and prolong the half-life of polyphenols [167]. Surface-modifying liposomes (e.g., PEGylated or ligand-targeted) also improve intestinal absorption and tissue targeting, as well as reduce systemic toxicity. Because of their biocompatibility and versatility, the liposome system has significant potential as a carrier for polyphenols for use in the pharmaceutical, nutraceutical, and functional food industries.

There is evidence that co-encapsulating EGCG and quercetin in liposomes improves their stability and functional performance. These optimized liposomes (approximately 111 nm) exhibited good physicochemical stability and satisfactory encapsulation efficiencies of both polyphenols and provided a synergistic antioxidant activity when compared to the individual polyphenols [156].

Trans-resveratrol is an important bioactive polyphenol known for antioxidant, anti-inflammatory, cardioprotective, neuroprotective, and anticancer activities [168]. In another study, resveratrol-loaded liposomes were developed to overcome stability and bioavailability limitations for oral cancer prevention and therapy. Optimized liposomes showed high biocompatibility, potent antioxidant activity, and efficient cellular uptake, with vitamin C enhancing antioxidant effects [154]. Encapsulation of rutin in liposomes and incorporation into HPMC-based edible films improved film physical properties, including flexibility, thickness, and color, as the particle size was reduced to ~106 nm with 89% encapsulation efficiency [155]. Liposome-loaded films exhibited slower rutin release compared with free rutin, providing a controlled delivery of antioxidants. This approach enhances the functionality of edible films, offering potential for gradual antioxidant release and extended food shelf life [155].

In another study, researchers developed curcumin-loaded liposome nanoparticles (CLLNs) via thin-film hydration and sonication to boost curcumin’s poor solubility and bioavailability for anti-inflammatory and anti-cancer uses. CLLNs showed ~250 nm size, −32 mV zeta potential, 75% encapsulation efficiency, 3% drug loading, and sustained release (~70% by 72 h), indicating stability and controlled delivery potential, though in vivo studies are needed [156].

### 7.3. Solid Lipid Nanoparticles

Solid lipid nanoparticles (SLNs) are biocompatible and biodegradable submicron-sized carriers composed of lipids that remain solid at body temperature. SLNs are being researched to increase the solubility, stability, and oral bioavailability of polyphenols such as curcumin, resveratrol, quercetin, and EGCG that have poor aqueous solubility and have high metabolic breakdown rates [157]. Encapsulation in SLNs protects polyphenols from being chemically degraded and from being degraded in the gastrointestinal tract, allows for controlled or sustained release, and can enhance cellular uptake of the polyphenols or target them to specific tissues. Also, SLNs provide improved advantages vs. traditional lipid-based carriers, like low toxicity, high physical stability, and scalability and hold great potential for use as carriers of nutraceuticals and pharmaceuticals or for functional food applications [169].

In an effort to enhance the efficacy of treating breast cancer, a novel type of solid lipid nanoparticle (TPGS-Res-SLN) was synthesized that consists of TPGS and resveratrol using a method called solvent injection. These nanoparticles, which have a zeta potential of approximately −25.6 mV and contain 32.4% resveratrol by weight, were able to enhance the uptake by the cells, induce mitochondrial dysfunction and promote apoptosis in chemoresistant SKBR3/PR (a type of breast cancer cell line) cells, while being significantly better at inhibiting the migration and invasion than free resveratrol. In vivo studies in an SKBR3/PR xenograft model demonstrated that TPGS-Res-SLNs exhibited enhanced antitumor efficacy, thus indicating that TPGS-Res-SLNs are a potentially effective way of overcoming multidrug resistance for the treatment of breast cancer [169].

Ramalingam et al. (2016) [158] developed resveratrol-loaded solid lipid nanoparticles (SLNs) surface-modified with N-trimethyl chitosan–grafted palmitic acid (TMC-g-PA) to overcome poor oral bioavailability, low solubility, and instability of resveratrol. The TMC-g-PA SLNs demonstrated enhanced stability in gastric conditions, sustained intestinal release, and a 3.8-fold increase in oral bioavailability compared with resveratrol suspension. These findings suggest that TMC-g-PA SLNs are a promising oral delivery system for improving the therapeutic potential of resveratrol [158].

### 7.4. Nanostructured Lipid Carriers (NLC)

In a study, epigallocatechin gallate–phospholipid complex–loaded nanostructured lipid carriers (EGCG-PC-NLCs) were developed to overcome the poor bioavailability, instability, and toxicity of EGCG for rheumatoid arthritis therapy. The optimized NLCs (~160 nm, narrow PDI, negative zeta potential) enhanced solubility, provided sustained drug release for 24 h, and improved intestinal permeability while reducing toxicity. In vitro, ex vivo, and in vivo evaluations demonstrated improved anti-rheumatic efficacy, lymphatic uptake, and prolonged systemic exposure, highlighting EGCG-PC-NLCs as a safer and more effective delivery strategy for RA management [170].

### 7.5. Nanoemulsions/Microemulsions

Nanoemulsions, also known as microemulsions, are stable, kinetically stable, submicron-sized (approximately 20 to 200 nm) dispersions of oil in water (or water in oil). These are ideal vehicles for use in delivering polyphenolic compounds that are known to have poor solubility, stability, and bioavailability. Polyphenolic compounds (such as curcumin, resveratrol, quercetin, and EGCG) can be encapsulated in nanoemulsions to increase their dispersibility in aqueous solutions, improve their chemical stabilities, and increase the amount that is absorbed when consumed orally by the body. The encapsulation of polyphenolic compounds in nanoemulsions will also mask the taste and/or bitterness associated with them [113].

Nam et al., 2024 [159] developed a stable oil-in-water EGCG nanoemulsion using lecithin, pectin, and gallic acid to overcome EGCG instability and bitterness. The optimized system showed high encapsulation efficiency (88.9%), small droplet size (~169 nm), low PDI, and long-term stability, with enhanced antioxidant activity and effective browning inhibition [159].

In another study done by Kotta et al., 2021 [160] resveratrol nanoemulsion was formulated via ultrasonication using response surface methodology, with coconut oil, Pluronic-P107, and Cremophor EL. These were further optimized with respect to oil/surfactant concentrations, ultrasonication time, intensity, and power to achieve small globule size, low PDI, and suitable zeta potential. The optimized formulation showed superior in vitro release in pH 6.8 buffer and permeation across goat nasal mucosa compared to controls and demonstrated high brain targeting efficiency upon intranasal administration (2 mg/kg) in rats [160].

Sharma et al., 2020 [161] developed a TPGS-loaded rutin nanoemulsion to mitigate oxidative stress–induced neurodegeneration in a rat model of Parkinson’s disease. Oral administration significantly enhanced rutin bioavailability (1.8-fold AUC and 1.9-fold Cmax) and produced superior pharmacodynamic outcomes, including improved motor behavior, reduced catalepsy, and restoration of antioxidant markers (↑GSH, ↑SOD, ↓MDA) compared with free rutin [161].

Mahadev et al., 2022 [162] developed ultrasonically assisted quercetin nanoemulsion (Que-NE) using ethyl oleate, Tween 20, and Labrasol, optimized via Box–Behnken design to achieve 125.51 nm droplet size, 0.215 PDI, and 87.04% entrapment efficiency at 9% Smix, 25% amplitude, and 2.5 min sonication. The stable, spherical formulation (confirmed by TEM) showed superior release, enhanced oral bioavailability over pure quercetin, and remained stable at 5–40 °C for 45 days. In streptozotocin-induced diabetic rats, Que-NE effectively managed body weight, blood glucose, lipid profile, tissue injury markers, and protected pancreatic β-cells and hepatocytes, positioning it as a promising antidiabetic therapy [162].

### 7.6. Solid Dispersions

The use of solid dispersions (SD’s) is a unique new way to create new formulations with poorly water-soluble compounds within a hydrophilic polymer matrix so that the poorly water-soluble compounds will have improved solubility, dissolution rates, and improved oral bioavailability. For instance, curcumin, quercetin, and resveratrol are examples of polyphenols that have low aqueous solubility, are metabolized rapidly, and are poorly absorbed systemically as a consequence of these three factors, and thus are not able to deliver their therapeutic potential. However, when polyphenols are incorporated into solid dispersions with polymers such as polyvinylpyrrolidone (PVP), polyethylene glycol (PEG), or hydroxypropyl methyl cellulose (HPMC), the polyphenols will remain in an amorphous state, will not crystallize, and thus will have a greater degree of stability and bioactivity than if they were not in an amorphous state or had crystallized [163]. Polyphenol solid dispersions can be prepared using solvent evaporation methods, hot-melt extrusion, or spray drying, and have been found to produce a greater antioxidant, anti-inflammatory, and anticancer effect from in vitro and in vivo studies of polyphenols.

To improve on issues of solubility and dissolution, solid dispersion (SD) methods are a popular way of converting crystalline forms of quercetin to an amorphous form in a hydrophilic polymer (e.g., PVP K30) and have been found to dramatically improve solubilization and dissolution rates of quercetin. For example, quercetin spray dried SD with PVP showed improved solubility and an increase in the percentage of dissolved quercetin (≈95%) after 120 min compared to the pure quercetin or physical mixture due to the fact that the presence of the polymer led to both amorphous state of quercetin and greater quercetin-polymer interactions [164]. Other types of solid dispersions, either with cellulose or cellulose derivatives, PVP also exhibited increased intestinal quercetin concentrations compared to both pure quercetin and physical mixture, on average by about 18 times as regards solution concentrations at intestinal pH, suggesting a significant increase in bioavailability [164].

Ha et al. (2021) [165] formulated amorphous solid dispersions of trans-resveratrol using Eudragit E/HCl to improve dissolution and oral bioavailability. The optimized formulation (resveratrol/polymer 10:90) effectively inhibited drug precipitation at gastric pH via the formation of polymeric micelles, providing prolonged supersaturation for 48 h. In vivo studies in rats indicated a significant increase in absorption with an absolute oral bioavailability of ~40%, demonstrating the potential for using Eudragit E/HCl as a carrier for the delivery of resveratrol [165].

A comparative evaluation of the strategies discussed above indicates that improving bioavailability remains one of the most critical determinants of successful clinical translation of polyphenols. Although conventional approaches such as co-administration with absorption enhancers and structural modification have shown benefits, nanotechnology-based delivery systems consistently demonstrate superior performance in enhancing solubility, stability, gastrointestinal absorption, and systemic exposure. Lipid-based carriers, polymeric nanoparticles, phytosomes, liposomes, and nanoemulsions have repeatedly been shown to improve pharmacokinetic profiles while simultaneously enhancing therapeutic efficacy in preclinical cancer models. Importantly, the literature suggests that the future success of polyphenol-based therapeutics will depend not only on the intrinsic biological activity of these compounds but also on the development of optimized delivery platforms capable of overcoming pharmacokinetic limitations and enabling targeted delivery to tumor tissues.

## 8. Preclinical and Clinical Studies of Polyphenols

Polyphenolic compounds such as curcumin, resveratrol, quercetin, EGCG and rutin exhibit potent anticancer properties based on the modulation of oxidative stress, inflammation, apoptosis, cell cycle arrest, angiogenesis and metastasis [166,167]. Studies using both in vitro and animal models consistently report inhibition of oncogenic signaling pathways (e.g., NF-κB, PI3K/Akt, STAT3, EGFR, VEGF, Bcl-2 family proteins), resulting in decreased tumor growth and increased chemosensitivity [171,172]. A summary of the anticancer efficacy of bioflavonoids in various animal models, along with mechanistic and therapeutic endpoints, can be found in Table 8.

Although preclinical studies consistently demonstrate the strong anticancer potential of polyphenols, their clinical translation has been constrained by poor oral bioavailability, rapid metabolism, low aqueous solubility, and extensive first-pass clearance, which collectively limit achievable systemic exposure. As summarized in Table 7, early clinical trials of major compounds such as curcumin (e.g., NCT00027495, NCT01201694) and resveratrol (e.g., NCT00256334) confirm that circulating and tissue levels in humans are often substantially lower than those required for preclinical efficacy.

Accordingly, most completed studies have not demonstrated direct tumor regression but instead report biomarker-level and pathway-level modulation. For instance, curcumin trials (NCT03980509, NCT01917890) show alterations in Ki67, NF-κB, COX-2, and oxidative stress markers, while green tea catechin and quercetin studies (NCT00676780, NCT01912820) demonstrate regulation of c-Met signaling, PI3K/MAPK pathways, and epigenetic enzymes such as DNMT1. In parallel, dietary and microbiome-associated interventions (NCT01916239, NCT03994055) highlight that polyphenol activity in humans is strongly influenced by gut microbial metabolism and anti-inflammatory dietary context, with measurable effects on tumor microenvironment and treatment-related toxicity.

To overcome pharmacokinetic barriers, advanced delivery strategies including liposomes, nanoemulsions, polymeric nanoparticles, and phytosomal formulations have been developed, with early clinical evidence indicating improved systemic exposure and tolerability. Some studies also suggest enhanced biological effects when polyphenols are used as adjuncts to chemotherapy or radiotherapy, particularly in modulating inflammation and treatment-associated toxicity.

Overall, clinical trials summarized in Table 9 collectively demonstrate that polyphenols exert reproducible molecular and pathway-level effects in cancer patients, but their clinical impact remains largely limited to mechanistic and supportive endpoints. Future progress will require standardized high-bioavailability formulations and well-powered randomized trials designed to evaluate clinically meaningful outcomes such as progression-free and overall survival.

## 9. Current Challenges and Research Gaps

Preclinical studies have demonstrated that plant-derived polyphenols possess anticancer properties; however, translating those findings to clinical use has proven challenging due to multiple factors and impediments to effective development of anticancer agents [197].

### 9.1. Lack of Standardized Formulations

One significant factor affecting the use of polyphenols as a cancer therapy is the lack of standardized formulations. Polyphenol formulations differ significantly based on source, extraction methods, purity, chemical stability and dosage forms, causing variations in bioavailability and efficacy [198]. Various formulations (i.e., free, nanoformulated, phospholipid complexed, encapsulated) also lead to additional variations between studies, making comparative analysis nearly impossible, therefore restricting reproducibility, regulatory approval and large-scale use.

### 9.2. Poor Correlation Between Preclinical and Clinical Data

Although many studies have demonstrated robust in vitro and in vivo anticancer activity by polyphenols, studies have regularly failed to translate into demonstrable outcomes when evaluated clinically due to the use of non-physiological concentrations in preclinical models, oversimplified experimental conditions, and species-specific differences in metabolism. Additionally, the complex microenvironment of tumors and variability among humans are usually not modeled by experimental animals, thus leading to over-estimation of therapeutic efficacy during preclinical evaluations [199].

### 9.3. Limited Understanding of Long-Term Safety

Most current studies examine the short-term cancer effects associated with polyphenol consumption but not how long they remain safe or their long-term toxicity profiles. Chronic use, especially if high doses are administered or if there are advanced delivery systems, may have side effects; cause metabolic interactions; and be pro-oxidant [198,200]. The safety of polyphenols, similarly, must be evaluated for their interactions with conventional anticancer medications (chemotherapeutics) as well as for their modulation of cellular signaling pathways over time.

### 9.4. Need for Better Bioavailability–Efficacy Correlation

The most important data gap is in establishing how improved bioavailability corresponds directly to anticancer efficacy. Advanced delivery strategies can dramatically increase the systemic exposure of polyphenols; however, increased bioavailability does not always equal better therapeutic outcomes [199]. Many key factors like tissue distribution/tissue retention, intracellular targeting, metabolic transformations and the production of active metabolites will ultimately determine the anticancer activity of polyphenols. Future research studies should include a combined effort of pharmacokinetic, pharmacodynamic and biomarker analysis to clearly establish the exposure-response relationship.

## 10. Future Perspectives

**Figure 5:**
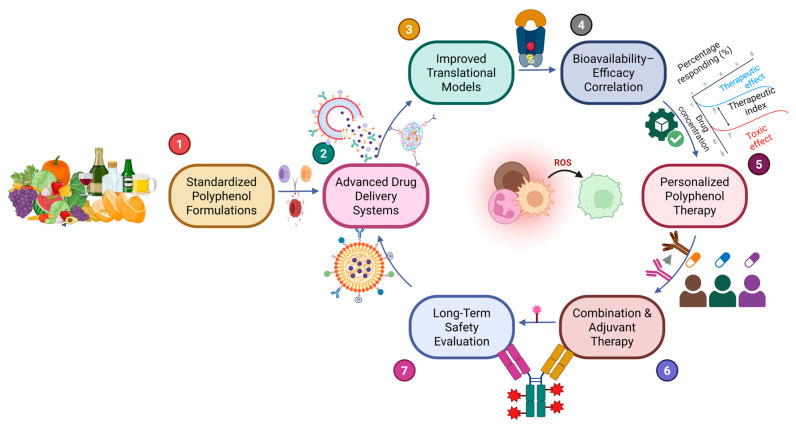
Future perspectives in the development of Plant-derived Polyphenols as Anticancer Therapeutics.

A multidimensional and translational research approach to the development of plant-derived polyphenols as effective anticancer agents needs to be taken in light of current limitations in formulation, bioavailability, efficacy, and safety research (Figure 5). The advancement of this area will require the ability to translate positive laboratory findings into clinically relevant outcomes. One of the primary objectives of this work includes establishing standardized methods for creating polyphenol formulations (i.e., consistent sourcing of raw materials; standardized extraction techniques; chemical composition analysis; standardized dosage forms) in order to facilitate reproducibility, compare results between studies, and translate the data from laboratory studies into clinical applications [200]. There will also be an emphasis on creating delivery systems that can be easily manufactured on a large scale and readily available to patients, such as using nanoparticles or phospholipid-based complexes [201].

Perhaps even more important than developing a common method to develop formulations of polyphenols is the need to develop a common preclinical testing model. Advanced preclinical models such as three-dimensional (3D) tumor models, organoids and patient-derived xenografts are able to demonstrate how well-suited a particular polyphenol is for either combination therapy or longer-term use by addressing the heterogeneity of human tumors and providing insight into the tumor microenvironment. Examples of how these advanced preclinical models could improve the predictivity of preclinical data and the relationship between lab efficacy and clinical outcomes are numerous.

One last important area to focus on is the long-term safety of the polyphenol under consideration. It will be important to conduct well-designed studies that evaluate the safety of the polyphenol when administered chronically, identify the maximum dose of the polyphenol that can be given without causing toxicity, define the metabolic fate of the polyphenol, and evaluate the interaction of the polyphenol with other drugs. In particular, it will be necessary to ensure that appropriate pharmacokinetic data are collected for the interaction of the polyphenol and chemotherapeutic agents administered concomitantly to avoid negative effects on drug disposition due to alteration of the activity of metabolizing enzymes or transporters [201].

Future efforts must be made toward developing a clear bioavailability–efficacy correlation by using pharmacokinetic, pharmacodynamic, and biomarker-related methods of analysis. By determining the distribution of the compounds within specific tissues, how they will specifically target certain cells, and how to enhance the pharmacological efficacy of various active metabolites, rational ways can be established to optimize both formulations and dosing regimens. Personalized approaches will improve outcomes as well; these approaches can account for genetic variability between individuals, the relative composition of gut microbiota, and variability in metabolism between people when creating therapeutic protocols.

Overall, addressing these future directions will be key to changing plant-derived polyphenols from promising bioactive molecules to evidence-based potential anticancer pharmaceuticals in clinical practice.

## 11. Conclusions

This review demonstrates that the anticancer efficacy of plant-derived polyphenols is not attributable to a single mechanism but rather to coordinated modulation of multiple oncogenic pathways. Despite differences in chemical structure, most polyphenols exert their anticancer effects through modulation of a common set of molecular targets and signaling pathways, particularly PI3K/Akt/mTOR, NF-κB, MAPK, STAT3, p53, and Wnt/β-catenin signaling pathways, resulting in suppression of proliferation, induction of apoptosis and autophagy, inhibition of angiogenesis and metastasis, and modulation of inflammatory and immune responses. A notable trend emerging from the reviewed studies is that flavonoids, particularly quercetin, apigenin, genistein, and kaempferol, demonstrate the broadest spectrum of molecular activities, whereas stilbenes such as resveratrol exhibit strong pleiotropic effects on tumor metabolism, oxidative stress, and cellular signaling. Phenolic acids and lignans, although comparatively less investigated, also display significant anticancer efficacy through regulation of redox balance, apoptosis, and oncogenic signaling pathways. Collectively, these findings suggest that the therapeutic value of polyphenols lies in their multitargeted mode of action, which may be particularly advantageous in overcoming the molecular complexity and heterogeneity of cancer.

However, a recurring observation throughout the literature is that promising preclinical activity does not consistently translate into clinical benefit because of poor aqueous solubility, limited bioavailability, rapid metabolism, and pharmacokinetic variability. Consequently, bioavailability rather than biological activity appears to be the principal barrier to clinical translation. Recent advances in nanoformulations, targeted delivery systems, phytosomes, and polymeric carriers have demonstrated substantial potential to overcome these limitations and enhance therapeutic performance.

Overall, the evidence indicates that the future success of polyphenol-based anticancer therapeutics will depend on integrating mechanistic understanding with optimized delivery technologies and rigorous clinical validation. Well-designed clinical studies employing standardized formulations, biomarker-guided patient stratification, and pharmacokinetic–pharmacodynamic correlation analyses will be essential for translating the multitargeted anticancer potential of polyphenols into effective precision oncology interventions.

## Figures and Tables

**Table 1:** Molecular mechanisms underlying the anticancer activity of major polyphenols.

Anticancer Mechanism	Polyphenols	Key Molecular Targets	Major Outcome	Anticancer Mechanism
Modulation of Oxidative Stress and Cellular Redox Signaling	Curcumin, Resveratrol, Quercetin, EGCG	Nrf2, ROS, SOD, GPx, Catalase,	Reduced oxidative damage and tumor growth	[17,18]
		PI3K/AKT/mTOR, nuclear factor kappa B (NF-kB) and STAT3	Higher cytotoxic effect	
Apoptosis	Curcumin, Resveratrol, Apigenin, Quercetin	Bax, Bcl-2, Caspase-3, Caspase-9, p53	Programmed cancer cell death	[19,20,21,22,23,24,25]
	Naringenin	caspase-3, p53, and Bax, Bcl-2, Survivin	Induction of apoptosis	
	Hesperitin	(NF-kB, Bcl-2	Promoted apoptosis	
	Daidezin	BAK	exhibited pro-apoptotic	
Autophagy	Resveratrol, Curcumin, EGCG, Kaempferol	AMPK, mTOR, LC3-II, Beclin-1	Autophagic cell death and chemo-sensitization	[26,27,28,29]
Inhibition of Cell Proliferation and Cell Cycle Progression	Quercetin, Genistein, Curcumin, Apigenin, EGCG	Cyclin D1, CDKs, p21, p27, p53, PI3K/Akt, MAPK, STAT3, EGFR	Reduced cell proliferation	[30,31]
Suppression of Angiogenesis and Metastasis	Resveratrol, EGCG, Quercetin	VEGF, HIF-1α, PI3K/Akt, MAPK, STAT3,	Reduced endothelial cell proliferation and new blood vessel formation	[32,33]
	Apigenin	EMT, MMP-2, MMP-9	Reduced invasion and migration	
Modulation of Inflammatory and Immune Signaling Pathways	EGCG, Quercetin, Curcumin, Resveratrol	TNF-α, IL-6, NF-κB, STAT3, COX-2	Enhanced antitumor immunity	[34,35]
Epigenetic regulation	EGCG, Resveratrol, Genistein, Curcumin, Quercetin	DNMTs, HDACs, HATs, miRNAs	Reactivation of tumor suppressor genes	[36,37]

**Table 6:** Aqueous solubility of various polyphenols.

Polyphenols	Solubility	References
Resveratrol	30 µg/mL	[140]
Quercetin	0.3 µg/mL	[141]
Hesperetin	1.4 µg/mL	[142]
Naringenin	45 µg/mL	[142]
Genistein	0.81 µg/mL	[143]
Daidzein	8.215 µg/mL	[143]

**Table 7:** Comparative summary of nanocarrier and polymeric systems for enhancing the bioavailability and therapeutic efficacy of polyphenols.

Delivery Platform	Polyphenol	Carrier	Particle Size Characterization	Drug Loading	Therapeutic Outcome	Application	References
Polymeric Nanoparticles	Quercetin	Poly(lipoic acid) NPs	~185 nm	84.8% EE	↑Oral BA to 29% (vs. 0.19% crystalline); prolonged systemic exposure	Oral delivery enhancement	[149]
	Quercetin	PBCA NPs ± Polysorbate-80	~161–167 nm	~75–80% EE	2.38–4.93 fold ↑ BA; enhanced brain distribution	CNS targeting	[150]
	Resveratrol	Galactosylated PLGA NPs	~108 nm	High EE	~335% ↑ vs. suspension; improved intestinal transport	Targeted oral delivery	[151]
	EGCG	Melanin NPs	<100 nm	π–π interaction incorporation	Retained antioxidant & antibacterial activity	Antioxidant stabilization	[152]
Liposomes	EGCG + Quercetin	Co-encapsulated liposomes	~111 nm	Satisfactory EE	Synergistic antioxidant effect	Stability & antioxidant enhancement	[153]
	Resveratrol	Resveratrol-loaded liposomes	Nano-sized (optimized)	Not specified	Improved stability & bioavailability	Oral cancer therapy	[154]
	Rutin	Liposomes in HPMC edible films	~106 nm	89% EE	Controlled antioxidant release	Functional food films	[155]
	Curcumin	Curcumin liposomes (thin-film hydration)	~250 nm; −32 mV	75% EE	Sustained release; improved solubility	Anti-inflammatory & anticancer	[156]
Solid Lipid Nanoparticles (SLNs)	Resveratrol	TPGS–Res-SLNs	−25.6 mV (stable nanoformulation)	32.4% DL	Superior antitumor efficacy; MDR reversal	Breast cancer therapy	[157]
	Resveratrol	TMC-g-PA modified SLNs	Nano-sized; gastric stable	High incorporation	3.8-fold ↑ oral BA	Oral bioavailability enhancement	[158]
Nanoemulsions	EGCG	Lecithin + Pectin + Gallic acid	~169 nm; low PDI	88.9% EE	Improved stability; browning inhibition	Functional food	[159]
	Resveratrol	Coconut oil + Pluronic P107 + Cremophor EL	Small globule size; low PDI	Not specified	Enhanced nasal permeation & brain targeting	Intranasal brain delivery	[160]
	Rutin	TPGS nanoemulsion	Nano-sized	Not specified	↑ 1.8-fold AUC; ↑ 1.9-fold Cmax	Neuroprotection	[161]
	Quercetin	Ethyl oleate + Tween 20 + Labrasol	125 nm; PDI 0.215	87% EE	Enhanced oral BA; improved glycemic control	Antidiabetic therapy	[162]
Solid Dispersions	Quercetin	PVP K30	Amorphous conversion	—	~95% release (120 min)	Dissolution enhancement	[163]
	Quercetin	Cellulose derivatives + PVP	Improved intestinal solubilization	—	18-fold **↑** solution levels	Intestinal absorption enhancement	[164]
	Trans-resveratrol	Eudragit E/HCl	Maintained supersaturation (48 h)	10:90 drug: polymer	~40% absolute oral BA	Supersaturation stabilization	[165]

**Table 8:** Preclinical studies of polyphenols in various animal models.

Bioflavonoid/Compound	Animal Model & Dose	Biological Target/Mechanism	Primary Outcomes	Implications	Reference
Quercetin (Parkinson’s disease models)	Rodent PD models (rats/mice; 10–400 mg/kg, oral/IP)	Antioxidant; anti-inflammatory; antiapoptotic signaling	Improved motor function; reduced oxidative stress; decreased neuroinflammation and apoptosis	Supports neuroprotective potential in dopaminergic degeneration	[173]
Quercetin (Acute kidney injury)	Rodent AKI models (varied doses)	Reduced oxidative stress; modulation of inflammatory cytokines	↓ Blood urea nitrogen; ↓ serum creatinine; ↓ TNF-α/IL-1β; ↑ SOD/CAT activity	Demonstrates renoprotective effects via antioxidative and anti-inflammatory pathways	[174]
Quercetin (Alzheimer’s disease models)	Mouse/rat AD models (multiple dosing regimens)	Antioxidant; modulation of Aβ aggregation, tau phosphorylation, synaptic signaling	Improved cognition; reduced Aβ deposition; enhanced antioxidant enzymes	Consistent neuroprotective efficacy in AD models	[175]
Resveratrol (Oral cancer models)	Rodent oral cancer xenografts (≤100 mg/kg/day)	Induces apoptosis; inhibits Akt/mTOR and JAK2/STAT3 pathways; suppresses EMT and angiogenesis	Reduced tumor growth; increased apoptotic markers; decreased metastasis-related proteins	Strong multi-target anticancer effects in vivo	[176]
Flavonoids in Obesity Models (e.g., quercetin, naringenin, EGCG, genistein, apigenin)	Diet-induced obese rodents (varied doses)	Modulation of AMPK, PPARγ, JNK signaling; antioxidant and anti-inflammatory effects	Reduced body weight; improved glucose tolerance; enhanced insulin sensitivity; improved lipid profile	Highlights the anti-obesity and metabolic regulatory potential of flavonoids	[177]
Catechin/EGCG (Myocardial ischemia–reperfusion injury)	Rodent cardiac I/R models	Antioxidative; mitochondrial protection; antiapoptotic	Reduced oxidative stress; improved cardiac biomarkers; preserved myocardial function	Indicates cardioprotective effects of catechins	[178]
Daidzein (Isoflavonoid; myocardial injury models)	Rodent myocardial I/R injury models	Anti-inflammatory; NF-κB inhibition; reduced apoptosis and autophagy	Reduced TNF-α/IL-6; decreased caspase-3 activity; improved histopathology	Suggests cardioprotective and anti-inflammatory activity of isoflavones	[179]
Mixed Flavonoid Supplementation (Nanotoxicity studies)	Rodent models exposed to nanomaterials	Enhanced antioxidant defense; suppressed pro-inflammatory mediators	Increased SOD, CAT, GSH; decreased NO, TNF-α; reduced liver, kidney, and brain injury	Demonstrates broad organ-protective effects under oxidative stress conditions	[180]

**Table 9:** Clinical studies of polyphenol-based interventions in cancer prevention and metabolic disorders.

Polyphenol	Class	Cancer Type	Dose	Study Design	Key Findings	Clinical Significance	Clinical Trial ID	References
Resveratrol	Stilbene	Colorectal cancer	20–160 mg/day	Phase I open-label	Modulation of Wnt signaling and gene expression in colonic mucosa	Chemopreventive molecular activity in humans	NCT00256334	[181]
Resveratrol	Stilbene	LAM	250–1000 mg/day	Phase II open-label	VEGF-D modulation; safety confirmed	Adjunct safety with mTOR inhibition	NCT03253913	[182]
Resveratrol	Stilbene	PCOS	Micronized formulation	RCT	Improved metabolic and inflammatory markers	Indirect anticancer relevance via metabolic modulation	NCT01720459	[183]
Resveratrol	Stilbene	Gastrointestinal neuroendocrine tumors	5 g/day orally	Open-label interventional biological study	Increased Notch-1 activation and modulation of tumor biomarkers	Demonstrated mechanistic anticancer activity and tolerability of high-dose resveratrol	NCT01476592	[184]
Mixed polyphenols	Dietary phenolics	Breast cancer	Dietary intervention	Metabolomic RCT	Tissue detection of resveratrol metabolites	Confirms tumor bioavailability	NCT03482401	[185]
Quercetin + green tea	Flavonoids	Prostate cancer	Dietary flavonoids	Phase I	DNMT1 and COMT modulation	Epigenetic chemoprevention	NCT01912820	[186]
Quercetin	Flavonoid	Cancer-related cachexia and inflammation in advanced cancer patients	Oral quercetin supplementation	Interventional clinical study	Evaluation of anti-inflammatory and metabolic effects of quercetin in cancer-associated systemic inflammation	Potential supportive therapeutic role in reducing cachexia-associated inflammation and improving quality of life	NCT05680662	[187]
Quercetin	Flavonoid	Chronic hepatitis C-associated hepatocellular carcinoma risk	Oral quercetin	Phase I dose-escalation study	Evaluated safety, pharmacokinetics, and tyrosine kinase inhibition potential	Suggested chemopreventive and antiproliferative potential in liver cancer-associated conditions	NCT01538316	[188]
Genistein	Isoflavone	Prostate cancer	Soy isoflavone supplementation	Phase II randomized trial	Evaluated PSA kinetics and molecular biomarkers following genistein supplementation	Suggested potential role in delaying prostate cancer progression and modulating androgen-related pathways	NCT01985763	[189]
Genistein	Isoflavone	Breast and endometrial cancer prevention	Oral genistein twice daily for 84 days	Randomized double-blind placebo-controlled Phase I trial	Reduced DNA damage and modulated apoptosis- and estrogen-related biomarkers	Demonstrated chemopreventive potential and biological safety in postmenopausal women	NCT00099008	[190]
Genistein	Isoflavone	Bladder cancer	Genistein supplementation before surgery	Phase II presurgical trial	Modulated EGFR signaling and proliferation biomarkers in bladder tumor tissue	Suggested potential utility as a neoadjuvant chemopreventive agent	NCT00244933	[191]
EGCG (Polyphenon E)	Catechin	Prostate cancer	Oral extract	Phase II	↓ c-Met, PI3K/MAPK signaling	Multi-pathway inhibition	NCT00676780	[192]
Pomegranate polyphenols	Ellagitannins	Colorectal cancer	Extract	Phase I–II	Urolithin formation in tumor tissue	Microbiome-mediated anticancer effect	NCT01916239	[193]
Dietary polyphenols	Mixed diet	Cervical cancer	Anti-inflammatory diet	RCT	↓ inflammatory cytokines, ↓ GI toxicity	Supportive oncology benefit	NCT03994055	[194]
Chlorogenic acid	Hydroxycinnamate	Advanced cancers	IV escalation	Phase I	Safety + oxidative stress modulation	First-in-human systemic polyphenol use	NCT02728349	[195]
Anthocyanins (blueberry)	Flavonoids	NSCLC	Diet + docetaxel	Phase II	Chemotherapy sensitization (exploratory)	Adjunct anticancer potential	NCT01426620	[196]

## Data Availability

No new data were created or analyzed in this study. Data sharing is not applicable.
